# Meeting of College Faculties

**Published:** 1884-09

**Authors:** E. G. Betty


### PROCEEDINGS.

### Meeting of College Faculties.

REPORTED BY E. G. BETTY, D.D.S.

A number of gentlemen representing the majority of the dental colleges in the country met together at the Sturtevant House, in New York City, at 10 o'clock A. M., Monday, August 4th, 1884. In pursuance of the object of the meeting, Dr. Foster, of Baltimore, in order to get to business, nominated Prof. C. N. Pierce, of the Pennsylvania College, as temporary chairman. Dr. Foster was nominated for temporary secretary, but withdrew in favor of Dr. H. A. Smith, of the Ohio College. The names of the colleges represented were then called as follows:

Baltimore, represented by Profs. Winder and Foster; University of Michigan, Prof. Taft; University of Pennsylvania, Prof. Truman; Phila. College, Prof. Garrettson; Ohio College, Prof. H. A. Smith; Boston, Profs. Blodgett and Follett; Iowa, Prof. A. O. Hunt; Penn. College, Profs. Henry Leffmann and C. N. Pierce; New York, Profs. Abbott and Littig; California University Dental Department, by letter; Kansas City, by letter.

The Chair appointed as a Committee on Organization, Drs. Truman, Winder, and Taft. A recess of half an hour was taken to give the committee time to make their report. At the end of this time Dr. Taft read the report, which is as follows:

### CONSTITUTION.

NAME.

Sec. I. This body shall be called The National Association of Dental Faculties.

OBJECTS.

Sec. II. The objects of this Association shall be to promote the interests of dental education—securing more uniform methods, and elevating the standard.

OFFICERS.

Sec. III. The officers shall consist of a President, a Vice-President, Secretary, and Treasurer, who shall be elected by ballot and shall hold office till their successors are elected.

QUORUM.

Sec. IV. Two-thirds of the colleges belonging to this Association shall be necessary to constitute a quorum.

Sec. V. Any contemplated change involving the interest of the Dental Schools represented in this Association shall require one year's notice before final action is taken.

Sec. VI. Three members shall be elected, who, with the officers, shall constitute an Executive Committee, who shall have power to designate the time and place of the meetings, make preparation for the same and pass upon all candidates.

MEMBERSHIPS.

Sec. VII. Any reputable dental college may be represented in this body upon submitting to the Executive Committee satisfactory credentials, signing the constitution, conforming to the rules and regulations of this body, and paying such assessments as may be made.

Dr. Winder moved that the report be read again in sections, so that each could be passed upon by the meeting: carried. The 1st, 2nd, and 3rd sections were adopted in their order as read.

After some discussion upon the manner in which the colleges were to vote, it was settled that each one vote as a unit with this understanding. The 4th section was also adopted. When the 5th section was read there was some discussion on the point that before any important change be made involving the interests of the colleges that one year's notice be given and that the words "Dental Schools represented in this body" be substituted. The section was adopted as amended. In the 6th section, two members to be elected, was changed to *three* members to be elected by ballot, and the section adopted as amended. When the 7th section came up Dr. Harlan offered a verbal resolution to insert a clause providing for a membership fee. Dr. Taft moved to amend this by substituting an annual assessment to defray such expenses as might be incurred. Carried. It was then suggested that a committee on membership be appointed to pass upon candidates for admission to the Association. Dr. Foster said that the officers and members composing the Executive Committee were appointed to act in that capacity, as it was impractical to multiply committees. Carried. The report, as a whole, was then adopted, and the constitution signed by the following colleges:

Baltimore College of Dental Surgery, M. W. Foster, R. B. Winder, Dean; Pennsylvania College of Dental Surgery, C. N. Pierce, Henry Leffmann; Philadelphia Dental College, J. E. Garrettson; New York College of Dentistry, Frank Abbott, J. Bond Littig; Boston Dental College, J. A. Follett, Dean, Albert N. Blodgett; Dental College of the University of Michigan, J. Taft; University of Iowa, Dental Department, A. O. Hunt; Chicago College of Dental Surgery, A. W. Harlan, F. H. Gardiner; Dental Department of the University of Pennsylvania, James Truman; Ohio College of Dental Surgery, H. A. Smith.

The gentleman from Harvard, Dr. Fillebrown, could not sign the constitution, as he had no delegated authority to act for his college, but thought that the forming of the Association was a good thing and it met with his hearty approval. On motion of Prof. Taft it was decided that during the time between the annual meetings any College desiring admission can become a member by applying to the Executive Committee, presenting satisfactory credentials and conforming to the rules, etc., as provided in the constitution. A motion was then carried to go into the election of permanent officers, the following gentlemen being elected:

President, Prof. C. N. Pierce; Vice-President, Prof. R. B. Winder; Secretary, Prof. H. A. Smith; Treasurer, Prof. A. W. Harlan.

The three members elected to serve on the Executive Committee were Profs. Abbott, Truman, and Taft.

Dr. Winder offered a resolution that after the session of 1884–85, that the colleges announce that five years' practice will no longer be accepted as an equivalent for one course of lectures. Dr. Taft said that was somewhat indefinite, as it was necessary to state exactly how many years, if any, *would* be required. Dr. Winder said that his resolution was one of inquiry only, in order to see what the members thought about it. Dr. Truman moved as a substitute for Dr. Winder's resolution that two full years' attendance be required at college; this was carried with enthusiasm. Dr. Smith made a motion to the effect that a medical graduate be required to attend but one course in a dental college, provided that he passed an examination satisfactory to the faculty. Dr. Abbott would amend this by requiring that such graduate of medicine be required to spend one full year in a college infirmary instead of but one *course*, so that he would have at least this length of time to qualify himself for practice, the year to include the course of lectures in the college. Dr. Foster thought that the student's ability should determine the length of time he was to spend at college. Dr. Abbott thought that there should be a minimum of time required. Dr. Taft remarked that the student of average ability is to be the basis of consideration, and not the extremes to which reference has been made.. He has had most trouble with the medical men because they imagine their attaiments are superior, and are not therefore inclined to study and work so hard. The best way undoubtedly is to require time sufficient to teach thoroughly. Dr. Smith said that the resolution was indefinite, and that "In College Infirmary" be substituted. Dr. Abbott accepted this, and his amendment was carried. Dr. Smith's resolution then, as amended, was unanimously carried. Dr. Abbott offered a resolution recognizing one course of medicine as equivalent to one year's office pupilage, but requiring of such student the attendance upon two courses of dental lectures. This was carried.

Dr. Harlan offered a resolution to be added: "Provided that a candidate for graduation shall have attended two regular courses of lectures in separate years." Carried unanimously. Dr. Taft then offered the following: "*Resolved*, That we recommend that three years study of dentistry be required for graduation from a dental college." Dr. Follett moved to amend by substituting "required" instead of recommend; no second. The resolution carried. Dr. Taft then moved that a committee of three be appointed by the Chair to consider curriculum in the schools; the Chair then appointed Drs. Taft, Truman, and Winder.

Adjourned.

AFTERNOON SESSION.

Upon the meeting being called to order, Dr. Winder said that he thought it best for the Association to meet biennially. Dr. Taft expressed the opinion that it would be more advisable to meet again a year hence, as there would undoubtedly be much of the business of this meeting requiring modification and perfecting; Dr. Taft's amendment was carried. He also moved that an assessment of $2.00 per member be collected from each college to meet the expenses incurred. Carried. Bills amounting to $15.25 were ordered paid. Dr. Truman then moved a reconsideration of the action of the Association requiring medical gradution as an equivalent for one year's course in a dental college. Dr. Winder and Dr. Taft both spoke against this, and the question being put, the motion was defeated. Dr. Garrettson received instruction from his college in writing which he put in the form of a written resolution to this effect: *Resolved*, That the colleges of this Association receive into the senior class only such juniors as hold certificates of having passed a satisfactory examination in the studies of junior year; this certificate to be a pledge to any college to which they may apply that a previous term has been properly spent in the institution whence they come.

After considerable discussion on this resolution, it was referred to the Committee on Curriculum to be brought up again at Saratoga, August 5th.

Adjourned.

SARATOGA, August 5th.

The meeting was called to order by President Pierce; the minutes of the previous session were read and approved. Dr. Taft read a letter from the Dean of the Dental Department of the University of California. Dr. Abbott said it was impossible for California to be represented by a letter and have the power to vote as it is necessary for the colleges to be present in the persons of their authorized representatives and sign the constitution personally, as a letter or proxy could not be construed to constitute membership. The Chairman stated that the constitution made provision for colleges to become members, but not by proxy. Dr. Truman--Can't a college vote by proxy after it has become a member of the Association? The Chair said that no provision had been made for that, and that the point would require further legislation to determine it. Dr. Taft moved that Dr. Truman act as proxy for Philadelphia. The President said this could be done by common consent, and as no one offered any objection, the motion prevailed. Dr. Smith said that it was his impression that Dr. Garrettson's resolution was laid on the table at the last session. Dr. Gardiner said that it was laid on the table before the adjournment of the morning session, but that it was brought up in the afternoon and referred to the Committee on Curriculum. The Chair ruled that it could be brought up as new business. A motion to do this was carried, and Dr. Abbott moved that it again be referred to the committee to further consider it: carried. Dr. Taft moved its adoption, Dr. Truman favored its going back to the committee. Dr. Taft, the Chairman of the Committee on Curriculum, made his report:

### REPORT ON CURRICULUM.

### PRELIMINARY EXAMINATION.

Your committee recommend that a preliminary examination be required for entrance to our dental colleges. Such requirements shall include a good English education.

In case of any student failing to pass a satisfactory preliminary examination the other colleges of this Association shall be informed of the fact.

Dr. Abbott moved that it be accepted: carried. Dr. Abbott said that as he understood it, the report contemplated action a year hence, and in view of that he moved that the report be adopted: carried. Dr. Truman said that he thought that part relating to a graded course was a recommendation, and could not see how the schools could change their order to conform with the provisions of the report, and had doubts about the practicability of the change. Dr. Darby said that we could recommend the adoption to the schools, and at all events it amounted only to a transposition of studies, and the thing is not impossible. Dr. Foster said that something was misunderstood, it was not intended to prohibit operative dentistry during the first year. He also thought it best that the deans be notified so that students would be prevented from going from one college to another, as an examination at one institution was enough to determine whether the student was fit or not. Dr. Hunt said that usually it is the custom in institutions to accept literary degrees without question. Dr. Gardiner asked if the preliminary examination was to be in writing. The President said that it was not specifically stated. Dr. Gardiner—It ought to be. Dr. Truman thought that such an examination should be a test of general information. Dr. Taft suggested an amendment so as to read "a reasonable knowledge of numbers, geography, and history." *Dr. Foster* said that when the subject was up before, opinion was so divided that we stopped at plain "English language." As Dr. Taft remarked, a candidate, though generally intelligent, might be deficient in some particular branches and yet be fit; the examining boards are the best judges of the capabilities of the candidates appearing before them. Dr. Abbott thought that the examination should be in writing. A simple statement of an examination was sufficient without specifying upon what subjects the examination would be made. Dr. Taft said that all he insisted upon was that the candidate possess such general knowledge as is usually taught in the schools, and certainly we have a right to expect that he shall have some training upon which to base his study of dentistry. Dr. Foster—Don't an ordinary English education comprise the studies enumerated? Dr. Taft—What I

mean is, an ordinary English education in the sense the term is generally understood. Dr. Foster moved an amendment to read "ordinary English education." Dr. Follett said that we might, and ought to, advance a little on what is called an ordinary English education. The candidate certainly ought to know numbers, geography, and history, but I would limit the latter term, as it might be construed to mean the entire history of the world from pre-historic times down to the present day. Dr. Taft—That point is modified in the report by the word "reasonable." Dr. Follett said that if a student did not pass a satisfactory examination, would the other colleges accept the decision? Is it justice to the student if another college refuse him? Dr. Smith said that the student might come back afterwards and be prepared to make his examination satisfactorily. It is best not to be unjust to the student as he might be acceptable to one college and not to another. Dr. Follett said that the clause should be so arranged as to *mean* something. If he studies for a year and qualifies himself he is entitled to try the examination again. Dr. Foster—The object of the report is that if a student applies to a college and is found incompetent that the other deans be notified. Any dean's notification to this effect is ample evidence that the applicant was not competent at that time, and as he could not enter that college then, neither could he enter another immediately afterward. Dr. Follett expressed the hope that the examination would be made in writing in part at least. Dr. Darby said that it was possible for the student to go from one school to another very rapidly and represent that he had never been examined, and this makes it possible for the colleges to fall into error if the deans are not notified at once of such student having failed to show himself capable. Dr. Foster said that examinations may or may not be severe, and should one college refuse him and another receive him into its classes, the latter shoulders all the discredit for having accepted an incompetent, while the other would assume great virtue and honor for the action it had taken. *Dr. Abbott* said that certain days could be specified upon which examinations might be held in order that all anticipated trouble might be avoided. Dr. Truman thought there were practical difficulties in the way of this course, as the dean has all that he can do to receive students from the middle of September to the same time in the following month without adding to his many duties by putting applicants through preliminary examinations. This would delay matters very materially, and he did not see what disposition could be made of those who had passed in the meanwhile. Dr. Smith—I think we are attempting entirely too much at the present meeting of this Association, as we are not ready to say just what the examinations shall consist of. It is advisable to wait until we learn what action has been taken by the American Medical College Association, as we are yet ignorant as to just what we want to do. It is much the wiser course to go slowly and with care than to adopt faulty methods that will undoubtedly have to be changed. Dr. Gardiner mentioned a college in which the examination papers of the preliminary examination were kept on file just the same as those of the final examination. Dr. Follett said that if his college refused an applicant, would the other colleges do the same, for if they would not, what would be the use of any notifications? Dr. Taft said that it was unlikely that more than two or three such cases would occur in a year's time. At any rate, when the uniform action of the colleges becomes known, as it undoubtedly will in a short time, the candidates will prepare themselves to pass the examination; six or eight hours is amply sufficient to examine half a hundred students and have the examination in writing at that. Dr. Follett—The report reads "shall" report. Dr. Taft—That was changed to read "may." Dr. H. A. Smith said that as the Chair had made no remarks on the subject, he would like to hear from him. Dr. Pierce said that he had been engaged in examining students for medical beneficiaries. The application of the candidate is always in his own hand-writing. They may also send credentials from the teachers in the schools they attended. The pastor, also, usually gives a certificate as to the student's general character. These examinations number about ten or twelve per annum. It seems to me that it is impossible either to specify questions or appoint days on which the preliminary examination is to be held. The examination for admission may in many cases be conducted by correspondence, thus saving considerable time. Dr. Smith—I think that literary graduates need no examination. Dr. Taft—The diplomas of a reputable institution are always recognized. The report may be changed to include this point, but it was tacitly understood. Dr. H. A. Smith moved to amend by inserting the clause: carried by common consent. The first part of the report as amended was carried unanimously. Dr. Taft then read the 2nd section of his report. Dr. Truman moved to adjourn and think over it till to-morrow. Dr. Foster did not think it of sufficient importance for that. Dr. Smith said he did not wish to be considered presumptuous, but "why not strike out specification and insert graded course."

Adjourned to meet August 6th, 4:30 p.m.

SARATOGA, Aug. 6th, 1884.

Dr. Pierce called the meeting to order, and the reading of the minutes being dispensed with, Dr. Abbott offered the following resolution:

*Resolved*, That we agree to adopt a graded course of instruction and an intermediate examination, which course of instruction and examination shall be conducted as the faculties of the different colleges represented in this Association may deem proper.

It being understood that it was a substitute for that portion relating to the graded course, Dr. Truman moved its adoption: carried unanimously. Dr. Hunt—If certificates are to be issued, is there to be any change in the methods of issuing tickets? Must the student not have a certificate as evidence of his examination?

*Dr. Abbott* moved that all students shall have a certificate from the school in which they were examined, which certificate shall be demanded by other colleges before admission. Dr. Hunt thought the Executive Committee should be instructed to prepare blank certificates to be filled up with those subjects upon which examination has been had, and be a plain statement of what the examination was. Dr. Darby—It is superfluous, as the certificate would always be demanded. *Dr. Abbott*—The matter should be referred to a committee to report upon next year. The resolution was carried unanimously. Dr. Taft made a further report from the Committee on Curriculum :

CURRICULUM.

We recommend that the following subjects and arrangements be adopted by the colleges of this Association, viz :

FIRST YEAR.

Anatomy with Dissections, Physiology, Histology, Chemistry Didactic and Practical, Mechanical Dentistry.

SECOND YEAR.

Review of the Junior Year Studies, Pathology, Surgery, Materia Medica, Therapeutics, and Operative Dentistry.

Dr. Truman moved that "a review of junior year studies" be stricken out. Dr. Abbott moved the adoption of the report as a recommendation. Carried. Dr. Foster moved that a committee be appointed to confer with the National Association of State Examiners to let them know what has been done by this Association. Dr. Taft—This can be done, and I have no doubt that Association will recognize the action of this body and discriminate accordingly.

Dr. Pierce said the committee ought to be furnished with official copies of the resolutions passed by this body. The resolution was carried, and the Chair appointed Drs. Foster, Abbott, and Hunt. Dr. Truman moved that the Secretary prepare printed copies of them and send them to the schools represented; Dr. Hunt suggested that the official results also be added. Carried unanimously. The Association then adjourned to meet a year from this time.

